# Completion and predictors of maternity continuum of care among women in the post-partum period in Gedeb district, southern Ethiopia: A community based cross-sectional study

**DOI:** 10.1371/journal.pone.0303380

**Published:** 2024-06-17

**Authors:** Gemechu Alemayehu, Simon Birhanu, Afework Alemayehu, Teshale Mulatu

**Affiliations:** 1 Department of Midwifery, College of Medicine and Health Sciences, Wachemo University, Hossana, Ethiopia; 2 College of Health Science, Debark University, Debark, Ethiopia; 3 School of Nursing and Midwifery, College of Health and Medical Science, Haramaya University, Harar, Ethiopia; Wollega University, ETHIOPIA

## Abstract

**Introduction:**

Maternal and neonatal complications related to pregnancy and childbirth pose a significant risk of morbidity and mortality to both the mother and the child. Despite its benefits in reducing maternal and neonatal mortality and morbidity associated with pregnancy and childbirth, the majority of Ethiopian mothers were dropped from the maternal continuum of care. Furthermore, there is a dearth of data regarding the status of the maternal continuum of care and its underlying factors in southern Ethiopia.

**Objective:**

This study aimed to assess the completion of the maternity continuum of care and its predictors among postpartum women who had given birth in the previous six months in the Gedeb district of Gedio Zone, southern Ethiopia.

**Methods:**

A community-based cross-sectional survey was conducted among 625 postpartum women selected by simple random sampling from June 1 to 30, 2022. The data was collected through face-to-face interviews using pretested, structured questionnaires. The association between the explanatory variables and the maternity continuum of care was examined using bivariate and multivariable logistic regression models. The adjusted odds ratio (AOR) with a 95% confidence interval was employed to measure the strength of association and the level of significance was set at p<0.05.

**Results:**

In this study, only 32.00% (95% CI: 28.45, 35.77) of the women completed the maternal continuum of care. Attending primary education (AOR = 2.09; 95% CI: 1.23, 3.55), secondary and above education (AOR = 1.97; 95% CI: 1.01, 3.87), receiving counseling during ANC (AOR = 1.89; 95% CI: 1.22, 2.92), being well prepared for birth and complications readiness (AOR = 4.13; 95% CI: 2.23, 7.62), and having good knowledge of pregnancy danger signs (AOR = 4.13; 95% CI: 2.60, 6.55) were all significantly associated with completing the maternity continuum of care.

**Conclusion:**

Nearly one-third of the women completed the maternity continuum of care. Enhancing women’s knowledge, offering counseling during prenatal visits, ensuring women’s awareness of pregnancy danger signs, and implementing health promotion programs targeted at enhancing birth preparedness and complications readiness for all are crucial.

## Introduction

Maternal mortality remains a major public health concern on a global scale. According to the WHO 2020 report, approximately 800 women die every day from avoidable complications related to pregnancy and childbirth. Of all maternal deaths globally, seventy percent occur in low- and middle-income countries, with Sub-Saharan Africa accounting for ninety-five percent of these deaths [1,2].

The target of sustainable development goal (SDG-3) is to bring the global maternal mortality ratio (MMR) down to 70 per 100,000 live births or less by 2030. However maternal mortality decreased by just 38% between 2000 and 2017. Despite advancements during the previous 20 years, over 295,000 maternal deaths occurred worldwide in 2017 as a result of inadequate or nonexistent maternity care during pregnancy and childbirth [2,3]. Ethiopia, with 412 maternal deaths per 100,000 live births, ranks among the top nations contributing more than 50% of all maternal deaths globally [4].

One efficient way to lower preventable maternal, neonatal, and child mortality is to implement a maternity continuum of care (COC). The "maternity continuum of care" refers to a range of health care services offered to women from pregnancy through the postpartum period, which comprises prenatal care, safe delivery services, and postnatal care [5,6]. It is estimated that 80% of maternal mortality and 66% of newborn deaths worldwide could be prevented if women had access to high-quality and efficient maternal healthcare services, during pregnancy, childbirth, and the first week of postpartum [7,8].

Although the continuum of maternal care is an effective strategy for enhancing the health of mothers and their newborns, the continuum of maternal care has been underutilized in low- and middle-income countries and sub-Saharan African countries. According to a multicounty systematic review report, only one-fourth (25.0%) of the women in sub-Saharan Africa completed the continuum of care (COC) for maternal health [9,10].

In Ethiopia, the completion rate of the maternal continuum of care varies from 6.56 to 67.8% [11,12].

In 2016, the WHO increased ANC visits from four visits to eight contacts intended to lower perinatal mortality and enhance pregnancy outcomes. However, Ethiopia has not yet fully implemented it [13,14]. According to the 2019 Ethiopia Demographic Health Survey report, 48% of the women gave birth at a healthcare facility, 34% had a PNC check-up, and 43.0% of the women had four ANC visits [10]. These figures provide an overview of the percentage of women receiving care at each stage, but they are not tracking particular individuals through the COC (continuum of care).

Low and middle-income countries continued to face challenges of high dropout rates from maternity continuum of care due to inadequate resources, poor health information systems, and fragmented health service infrastructures. The risk of maternal and neonatal death in the area is increased by the high rates of dropout from the maternal continuum of care, which means that many mothers and their infants may not get vital life-saving treatments that are provided at every stage of the continuum of care path [9,11,12].

Studies indicated that several factors have been found to influence the completion of the maternity continuum of care. Educational attainment, place of residence, occupation, parity, distance from healthcare facilities, use of transportation, decision-making authority of the woman, early initiation of antenatal care, wealth index, access to media, knowledge of pregnancy danger signs, receiving health education on maternal healthcare services, planned pregnancy, place of delivery, and mode of delivery were some of the identified factors that affect the completion of maternity continuum of care [15–19].

The Ethiopian Ministry of Health developed the Strategic Health Sector Transformation Plan (HSTP-IV 2015/16–2019/20) to offer all Ethiopian mothers equitable and high-quality care. This plan calls for increasing the number of healthcare professionals with training in maternal and child health, investing in health infrastructure, and providing free maternity services at all levels [20].

Despite the efforts to enhance maternal health care services, only a few mothers in Ethiopia have completed the maternity continuum of care. Furthermore, there is a dearth of information regarding the status of the maternity continuum of care in southern Ethiopia and the factors that contribute to it. Thus, this study examined the completion and predictors of the maternity continuum of care among postpartum women who had given birth in the previous six months in the Gedeb district of Gedio Zone, southern Ethiopia.

## Methods and material

### Study design and setting

A community-based cross-sectional survey was carried out from June 1 to 30, 2022, among postpartum women who had given birth during the previous six months in Gedeb district, southern Ethiopia.

Gedeb district is one of the Gedio zone districts, in the Southern Nation Nationalities People’s Region (SNNPR), situated around 433 kilometers from the capital city (Addis Ababa). It has 12 kebeles (the lowest structural administrative unit in Ethiopia), 10 of which are rural and 2 of which are urban kebeles According to the Gedeb district Health Bureau report, the projected population of 2021 was around 252195; of those, 121194 were males and 131001 were females, and of those, 30131 (23%) were reproductive-age women. There is a primary hospital, 4 health centers, 5 private clinics, and 14 health posts in the Gedeb district [21]. All public health facilities provide maternal health care services.

### Population and eligibility

The source population for this study was all women who gave birth in the Gedeb district six months before the study, whereas those women who gave birth six months preceding the study in randomly selected kebeles were the study population. Those women who were critically ill, unable to respond and women who referred from another area after obtaining one of the maternity care services were excluded from the study.

### Sample size and sampling technique

The sample size for this study was computed using a single population proportion formula with the assumptions of proportion P = 0.47% [22], 95% confidence interval, 4% margin of error, and adding a 10% non-response rate, giving the final sample size of 628.

Gedeb district has a total of 12 kebeles. From 12 kebeles, six kebeles were selected by lottery methods. The calculated sample size was proportionally allocated to each selected kebele. As a sampling frame, the list of all the women in the chosen kebeles was taken from the family folder or registration book kept by the health extension workers at the nearby health post. After obtaining the mother’s address and house number, an interview was held at home. A simple random sampling technique was used to choose study participants. Only one woman was interviewed by chance if more than one eligible woman was in the selected households.

### Data collection tool and procedures

A pretested, structured questionnaire was used to collect data through face-to-face interviews. The data collection tool was developed by reviewing various works of literature [5,6,15–18,22–27]. The tool comprises socio-demographic characteristics, maternal and neonatal health related variables, and healthcare services related variables.

Six BSc midwives collected the data, and two senior BSc midwives supervised the entire procedure. The data collectors received one-day instruction on the objectives of the study, how to complete the questionnaire, and how to deal with respondents. The tool was pretested on 5% of the sample, who were not from the study district. Any necessary modifications were then made to the instrument. Supervisors reviewed the gathered data every day to ensure consistency and completeness.

### Data processing and analysis

Epi-data version 3.1 was used to code and input the data into the computer, and SPSS version 26 was used for analysis. Descriptive statistics were computed to describe the characteristics of the study participants. A binary logistic regression analysis was used to examine the association between the independent and dependent variables. For multi-variable logistic regression analysis, the explanatory variables that had a P-value of less than 0.25 in the bi-variable analysis were deemed suitable. The variance inflation factor (<10) was used to assess multicollinearity, and Hosmer-Lemeshow’s test (P>0.05) was used to assess model fitness. The direction and strength of association was explained using an adjusted odds ratio (AOR) with a 95% confidence interval and a P-value of less than 0.05 was considered significant in the final model.

### Measurement

The maternity continuum of care was considered complete if the women had four ANC visits or more, had SBA assistance during childbirth or delivery, and had at least one PNC check-up within six weeks after giving birth at a heath facility or home with trained healthcare providers. If the mother had missed any of this continuum of care, the continuum of care was regarded as "not completed" [6,22].

The women were classified as having a good knowledge of pregnancy danger signs if they identified at least two of the four main pregnancy danger signs; vaginal bleeding, severe headaches, blurred vision, and swelling of the face or leg. If not, they were considered to have poor knowledge [16,17].

The women were regarded as well-prepared if the women reported having employed five or more BPCR components. If not, they were classified as not well-prepared. The BPCR elements taken into consideration in this study were the birthplace, the birth attendant, the ability to recognize labor signs, the preparation of supplies needed for labor and delivery, emergency savings, preparation of emergency transportation, the availability of people to support the mother and child during and after the birth, and potential blood donors when needed [16,17].

### Ethical considerations

Ethical clearance for this study was obtained from Haramaya University, College of Health and Medical Sciences, Institutional Health Research Ethics Review Committee (IHRERC) with reference number IHRERC/090/2022. Informed verbal and written consent was directly obtained from all study participants (those who gave birth). All the study participants had experience with childbirth, were competent enough to give consent and capable of making decisions on their behalf. Confidentiality was maintained anonymously.

## Results

### Socio-demographic characteristics of participants

A total of 625 postpartum mothers included in the study yielding a 99.5% response rate. The majority of the participants, 466 (74.5%) were in the 20–30 age range with a mean age of 25.48 (SD ± 4.67) years. The majority of the respondents, 615 (98.4%) were married, and nearly two-thirds or 404 (64.6%) lived in rural areas. In terms of education, 256 (41.0%) of the husbands and 220 (35.2%) of the women had completed elementary school. Regarding employment, 370 (59.20%) were housewives, while 95 (15.20%) worked in agriculture (Table 1).

**Table 1:** Socio-demographic characteristics of postpartum women who gave birth in the last six months in Gedeb district, southern Ethiopia 2022 (n = 625).

Variable	Categories		Frequency (n)	Percent (%)
Age of mothers(years)	15–19		38	6.10
	20–24		239	38.20
	25–29		227	36.30
	30–34		81	13.00
	≥35		40	6.40
Residence	Urban		222	35.52
	Rural		403	64.48
Marital status	Married		615	98.40
	Single		10	1.60
Mothers educational status	Unable to read and write		217	34.75
	Able to read or write		78	12.48
	Primary education (1–8)		220	35.20
	Secondary (9–12) and colleges		110	17.60
Mothers’ occupation	Housewife		370	59.20
	Farmer		95	15.20
	Merchant.		90	14.40
	Government Employee		58	9.28
	*Others		12	1.92
Husbands’ education	Unable to read and write		73	11.70
	Able to read or write		105	16.80
	Primary education (1–8)		256	41.0
	secondary colleges (9–12)		96	15.40
	colleges and above		95	15.20
Husband’s occupation	Farmer		237	37.90
	Merchant		196	31.40
	Government Employee		85	13.60
	NGOs Employee		16	2.60
	Unemployed/Daily labourer		53	8.50
	Student		38	6.10
Exposure to media	TV/Radio	Yes	256	40.96
		No	369	59.04

*Others = daily laborer, NGOs, Student.

### Obstetrics and reproductive health characteristics

More than half (325; 52.0%) of the women were multi-parous, and most of 567 (90.7%) pregnancies were planned and wanted. Regarding the obstetric history of participants, 49(7.84%) of them had bad obstetric history of which 22 (44.9%) had a history of abortion, followed by IUFD/Stillbirth, 20 (40.80%).

Among all women 40(6.40%) had obstetric complications, of which pregnancy-induced hypertension accounts for more than half 22(55.0%) of pregnancy-related complications (Table 2).

**Table 2:** Obstetrics and reproductive health characteristics of postpartum women who gave birth in the last six months in Gedeb district, southern Ethiopia 2022(n = 625).

Variable	Categories	Frequency (n)	Percent (%)
Parity	Prim-para	166	26.6
	Multiparous	325	52.0
	Grand multiparous	134	21.4
Pregnancy neediness	Planned	567	90.7
	Unplanned	58	9.30
Had bad obstetric history before index baby	Yes	49	7.8
	No	576	92.2
Types of bad obstetric history before index baby (n = 49)	Abortion	22	44.90
	IUFD/Stillbirth/	20	40.80
	early neonatal loss	7	14.30
Had obstetric complication	Yes	40	6.40
	No	585	93.70
Obstetric complication during pregnancy or delivery or PNC period (n = 40)	Pregnancy induced hypertension	22	55.00
	Antepartum haemorrhage	9	22.50
	Hyper emesis gravidarum	9	22.50
Mode of delivery	Spontaneous vaginal delivery	600	96.00
	Caesarean section or IVD	25	4.00

**NB**: IUFD = intrauterine fetal death, PNC = Postnatal care, IVD = Instrument assisted vaginal delivery.

### Maternal health care services utilization

Of all participants, more than three fourth, 486 (77.8%) had an ANC visit. More than half, 259 (53.23%) of the participants had four or more ANC visits, and the majority, 438 (90.1%), of them received tetanus toxoid injections during pregnancy.

Of all participants, 384 (61.44%) of them were well-prepared for delivery and 273 (56.17%) knew at least two obstetric danger signs. About three fifth, 372 (59.52%) of the participants gave birth at a health facility. Sudden onset of labor 124 (49.01%) and lack of transportation to reach health facilities 67 (26.48%) were the main reasons mentioned for not seeking delivery care services at a health facility. About two fifth, 254 (40.64%) of the participants had PNC checkup, of which 203 (80.31%) had a PNC checkup only once, and the majority, 173 (68.11%) received a PNC at hospitals (Table 3).

**Table 3:** Maternal health care services utilization among postpartum women who gave birth in the last six months in Gedeb district, southern Ethiopia, 2022.

Variable	Categories	Frequency (n)	Percent (%)
Attended ANC at health facility (n = 625)	Yes	486	77.8
	No	139	22.2
Number of ANC (n = 486)	One	30	6.17
	Two	77	15.84
	Three	120	24.69
	Four	259	53.29
Get counselled by health care provider (n = 625)	Yes	190	30.40
	No	435	69.60
Status of BPCR (n = 625)	Well prepared	384	61.44
	Not well prepared	241	38.56
Knowledge of pregnancy danger signs (n = 625)	Good knowledge	273	43.7
	Poor knowledge	352	56.3
Place of delivery (n = 625)	Health facility	372	59.52
	Home	253	40.48
Reason for non-utilization of institutional delivery for those who gave birth at home (n = 253)	Dislike health facility	38	15.2
	Delay of ambulance (lack of transportation for getting to health facilities)	67	26.48
	No road accesses.	24	9.49
	Sudden onset of labor	124	49.01
Accessed for PNC service	Yes	254	40.64
	No	371	59.36
Number of PNC visit	1 time	204	80.31
	2 times	47	18.50
	≥3 times	3	1.18
Place of PNC attended	Hospital	173	68.11
	Health centre	58	22.83
	Health post	19	7.48
	Own home	4	1.57

**NB**: ANC = Antenatal care, BPCR = birth preparedness and complication readiness, PNC = Postnatal care.

### Status of maternity continuum of care

**Fig 1:**
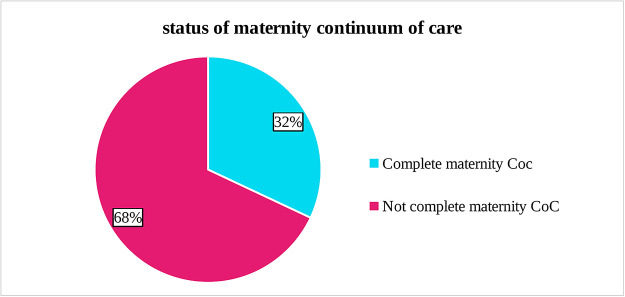
The status of maternity continuum of care among postpartum women who gave birth in the last six months in Gedeb district, southern Ethiopia, 2022 (n = 625).

Of all women, only 200 (32.00%) [95% CI: 28.45, 35.77] completed the maternity continuum of care (Fig 1).

### Predictors of maternity continuum of care

In the bivariate analysis: residence, maternal educational status, media exposure, travel time to a health facility, decision-making for seeking health care, obstetric complications during pregnancy, receiving counseling from a healthcare professional, BPCR (birth preparedness and complication readiness plan), and knowledge of pregnancy danger signs were associated with the maternity continuum of care at p<0.25. After controlling for confounders in the multivariable logistic regression; maternal educational status, getting counseling by a health provider, BPCR, and knowledge of pregnancy danger signs remained significantly associated with the maternity continuum of care.

The odds of completing maternity care were 2.09 times [AOR = 2.09; 95% CI (1.23, 3.55)] higher among those who completed their primary education and 1.97 times [AOR = 1.97; 95% CI (1.01, 3.87)] higher among those who completed their secondary school. Moreover, women who got ANC counseling from healthcare professionals had nearly double the odds of completing maternity care [AOR = 1.89; 95% CI: 1.22, 2.9] than women who did not get counseling from healthcare providers.

Furthermore, women with well-prepared birth plans and complication readiness had 4.12 times [AOR = 4.12; 95% CI (2.23, 7.62)] greater odds of completing a maternal continuum of care than their counterparts. In addition, women with good knowledge about pregnancy danger signs were 4.13 times [AOR = 4.13; 95% CI (2.60, 6.55)] more likely to complete the maternity continuum of care than their counterparts (Table 4).

**Table 4:** Predictors of maternity continuum of care among postpartum women who gave birth in the last six months in Gedeb district, southern Ethiopia 2022 (n = 625).

Variable	Maternity COC		COR (95% CI)	AOR (95% CI)	P-value
	Yes (%)	No (%)			
**Residence**					
Rural	94(23.33)	309(76.67)	1	1	
Urban	106(47.75)	116(52.25)	3.00(2.11, 4.26)	1.54(0.76, 3.09)	0.229
**Maternal educational status**					
Unable to read and write	39(17.97)	178(82.03)	1	1	
Able to read and write	18(23.08)	60 (76.92)	1.36(0.73, 2.57)	1.23(0.58, 2.60)	0.580
Primary education (1–8)	85(38.64)	135(61.36)	2.87(1.85, 4.46)	2.09(1.23, 3.55)*	0.006
Secondary, colleges & above	52(47.27)	58(52.73)	5.09(3.06, 8.47)	1.97(1.01, 3.87)*	0.047
**Exposed to media**					
No	96(26.02)	273(73.98)	1	1	
Yes	104(40.63)	152(59.37)	1.95(1.38, 2.74)	1.41(0.91, 2.16)	0.109
**Time to health facility**					
>30 minutes	84(23.33)	276(76.67)	1	1	
< = 30 minutes	116(43.77)	149(56.23)	2.55 (1.81, 3.61)	1.26(0.64, 2.45)	0.493
**Decision maker for the health care seeking**					
Family or husband only	81(26.64)	223(73.36)	1	1	
Women alone	119(37.07)	202(62.93)	1.62 (1.15, 2.27)	1.41(0.92, 2.16)	0.111
**Obstetric complication during pregnancy**					
No	177(22.29)	408(77.71)	1	1	
Yes	23(57.50)	17(42.50)	3.11(1.62, 5.98)	1.66(0.77, 3.57)	0.190
**Get counseling by health care provider**					
No	100(22.99)	335(77.01)	1	1	
Yes	100(52.63)	90(47.37)	3.72(2.59, 5.34)	1.89(1.22, 2.92)*	0.004
**Birth preparedness and complication readiness**					
Not Well prepared	17(7.05)	224(92.95)	1	1	
Well prepared	183(47.66)	201(52.34)	11.99(7.04,20.42)	4.12(2.23, 7.62)*	0.0001
**Knowledge of pregnancy danger signs**					
Poor knowledge	43(12.22)	309(87.78)	1	1	
Good knowledgeable	157(57.51)	116(42.49)	9.72 (6.52, 14.49)	4.13(2.60, 6.55)*	0.0001

*Significant at: P<0.05.

COR = crude odds ratio; AOR = adjusted odds ratio; CI = confidence interval; 1 = reference; BPCR = birth preparedness and complication readiness.

## Discussion

Reducing maternal, neonatal and child mortality can be achieved primarily through the implementation of the maternity continuum of care, which is an essential package of interventions that integrates maternal and child healthcare services. This study aimed to assess the completion and predictors of the maternal continuum of care among postpartum women who had given birth during the previous six months in the Gedeb district, southern Ethiopia.

The overall completion of the maternity continuum of care in this study was 32.00% (95% CI: 28.45, 35.77). The results showed that a significant proportion of women (68%) left the maternal continuum of care. Long distance to a health facility, lack of knowledge and exposure to the media, and lack of counseling by health care practitioners could be the causes of this high dropout rate from the maternal continuum of care as indicated in this study.

The level of maternity continuum of care in this study is in line with a study done in Southern Benin, 29.8% [25]. But, it was lower than the study findings from Ghana (66%) [26] and Egypt (50.4%) [27]. The disparities may result from difference in healthcare coverage, study settings, and respondent levels of education. Majority of respondents in previous studies were educated and lived in urban areas.

The result of this study is also lower than the study findings conducted in Northwest Ethiopia, 47% [23], east Gojjam, Ethiopia, 45% [15] and in southern Gonder, Ethiopia, 37.6%, [19]. The observed discrepancy could be due to regional variations in infrastructure as well as variations in the accessibility and availability of healthcare services. Another possible explanation could be attributed to the ANC’s cut point of inclusion criteria. In this study, ANC service utilization at least four times across the continuum of care was used to measure the completeness of COC in addition to delivery assisted by skilled personnel and PNC checkups received at least once from health care providers, whereas the pioneer studies used having at least one ANC visit across the continuum of care to measure the completeness of COC. Thus, women who had less than four ANC visits through the continuum of care in our study were considered as a dropout from pathway of maternity continuum of care, which may result in an underestimation of the maternity COC completion rate.

The proportion of women who completed maternity COC in this study was higher in comparison to studies carried out in Legambo district (11.2%) [6], Siyadebirena Wayu district (16.1%) [28], and multilevel analysis results from the Ethiopian Demographic and Health Survey (EDHS), 2016 (9.1%) [29]. The potential explanations for the observed discrepancies might be differences in the sample size, study area, participant sociodemographics, and time intervals between the investigations. For example, a large geographic area and sample was included in the multilevel analysis derived from the 2016 Ethiopian Demographic and Health Survey (EDHS).

This study’s findings showed that women’s educational status was positively associated with completing the maternity continuum of care. In comparison to women who did not attend formal education, those who attended primary and secondary education were about twice as likely to complete the maternal continuum of care. This result aligns with study conducted in Debre Berhan town, Central Ethiopia [16], northwest Ethiopia [20], and Zambia [30].

This could be because education increases women’s access to knowledge, changing their cognitive abilities, so they can comprehend health messages in the media more easily and improve their ability to communicate with healthcare providers in order to utilize available healthcare services. Education may also enable women to make decisions about household matters, including health care services [11,31]. This highlights the significance of education in ensuring the knowledge of mothers on the maternal continuum of care and enhancing their healthcare seeking behavior for themselves and their children.

In this study, women who received health education or counseling from a healthcare practitioner during ANC follow-up had 1.89 times greater odds of completing a maternal continuum of care than those who did not receive ANC counseling. This result is consistent with other research findings conducted in Ethiopia [20,23].

The reason for this might be that health education programs led by healthcare professionals raise women’s knowledge of the risks associated with receiving inadequate care during pregnancy and the necessity of receiving it; consequently, they are more likely to seek out maternal healthcare services. This highlighted the importance of counseling and maternal health education by skilled healthcare providers in increasing the uptake of maternal healthcare services across the continuum of care.

Furthermore, this study showed that completing the maternity continuum of care is significantly predicted by having a birth preparedness and complication readiness (BPCR) plan before labor. The likelihood of completing the maternity continuum of care was 4.12 times higher for women who were well prepared for the birth preparedness and complication readiness plan than for those who were not. This study finding is backed by study from Debre Berhan Town, Ethiopia [16].

This could be explained by the fact that knowledge about BPCR during prenatal care enables women to have a greater sense of social support for obtaining maternal health services as well as an appreciation for the significance of birth preparation and complication readiness plans This encouraged the women to seek out improved obstetric and infant care as well as timely treatment at health facility [32,33].

Moreover, this study showed that women with good knowledge of the warning signs of pregnancy were 4.13 times more likely to complete the maternity continuum of care than those with poor knowledge. This is consistent with studies done in west Gojjam [34], Debre Berhan [16], and the findings of EDHS 2016 in Ethiopia [11]. This finding is also in line with previous research conducted in Haramaya District, Eastern Ethiopia, which showed that women who were aware of the potential complications of their pregnancy were more likely to seek out maternal health services [35].

The possible explanation is that those women who are aware of the warning signals of pregnancy and its complications are driven to seek out medical care for their newborns as well as for themselves.

### Strength and limitations of the study

This study included both rural and urban kebeles which makes it representative and generalizable. Furthermore, the study’s community-based approach can give a precise picture of the problem. However, recall bias may be introduced during data collection. Furthermore, since the study was cross-sectional, it was not possible to establish the temporal relationship between variables.

## Conclusions

More than two third of the women were dropped out from the maternity continuum of care. Women’s education level, counseling from a health care provider, birth preparedness and complication readiness plans, and knowledge of pregnancy danger signs were important predictors of the maternity continuum of care. Counseling during ANC follow-up, ensuring knowledge of the women’s pregnancy danger signs, and health promotion programs aimed at improving birth preparedness and complication readiness are crucial to increasing the level of the maternity continuum of care. Furthermore, advanced studies to establish common tools to assess the maternity continuum of care and qualitative studies may be needed to explore barriers to the maternity continuum of care.

## Supporting information

S1 Dataset (SAV)

## Abbreviations

ANC: Antenatal Care
AOR: Adjusted Odds Ratio
BPCRs: Birth Preparedness and Complication Readiness Plan
*CI*: Confidence Interval
CoC: Continuum of Care
EDHS: Ethiopian Demographic Health Survey
PNC: Postnatal Care
SBA: Skilled Birth Attendants
WHO: World Health Organization
